# Study on the performance of emulsion explosives sensitized by an oxygen-generating M foaming agent

**DOI:** 10.1039/d5ra06873h

**Published:** 2025-10-21

**Authors:** Quan Wang, Kaiyan Lu, Yingkang Yao

**Affiliations:** a State Key Laboratory of Precision Blasting, Jianghan University Wuhan 430056 China wqaust@163.com; b Hubei Key Laboratory of Blasting Engineering, Jianghan University Wuhan 430056 China; c School of Safety Science and Engineering, Anhui University of Science and Technology Huainan P. R. China

## Abstract

To address the limitation of traditional nitrogen-sensitized emulsion explosives, which only produce a hot-spot effect without contributing energy, this study proposes an energy synergy enhancement strategy based on oxygen sensitization. By utilizing the oxygen generated from the reaction of an M foaming agent at varying concentrations to sensitize the emulsion explosives, with conventional emulsion explosives as a control group, the explosion performance was systematically evaluated through detonation velocity and air blast experiments. The influence on thermal decomposition properties was investigated *via* TG-DSC tests by fitting kinetic parameters. The results indicate that compared to the sodium nitrite control group, the mean detonation velocity of the M foaming agent-sensitized system increased from 4280 m s^−1^ to 4360 m s^−1^, reaching a peak of 4664 m s^−1^ at a 0.6% additive amount—an increase of 9%. The energy release characteristics exhibited significant concentration dependence: the peak overpressure (272.88 kPa) in air blast tests for the system with 0.6% M foaming agent was 81.5% higher than that of the 0.2% system, and the positive impulse increased by 62%. TG-DSC tests revealed that the peak thermal decomposition temperature of the M foaming agent-sensitized emulsion explosives generally shifted to higher temperatures with increasing additive amount, and the apparent activation energy also increased, indicating enhanced thermal stability. The chemical sensitization-energy synergy mechanism established in this study provides a novel approach for developing high-energy, high thermal stability emulsion explosives.

## Abstract 2

To address the limitation of traditional nitrogen-sensitized emulsion explosives, which only produce a hot-spot effect without contributing energy, this study proposes an energy synergy enhancement strategy based on oxygen sensitization.

## Graphical abstract

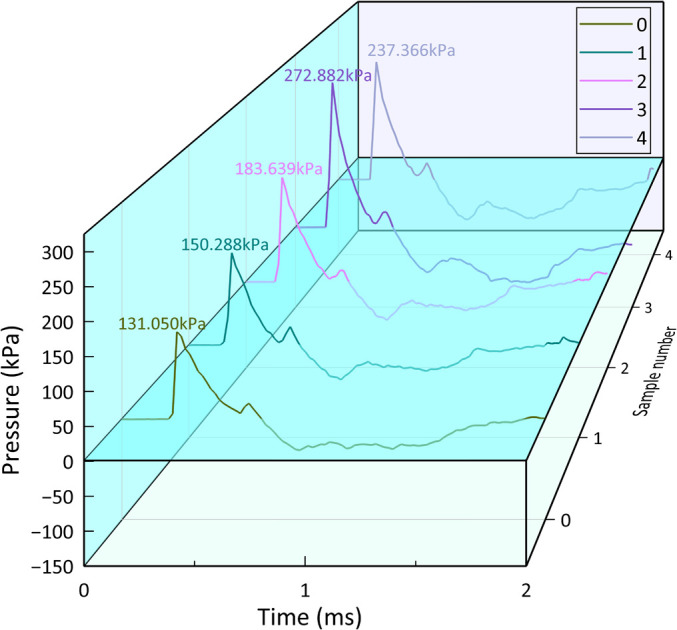

## 1 Introduction

Emulsion explosives are a class of industrial explosives characterized by a water-in-oil (W/O) structure. They are typically formulated by emulsifying an aqueous oxidizer salt solution with a fuel oil phase under high temperature and intense shear stress, followed by a sensitization process to attain detonability.^1–3^ Due to their excellent explosive performance, safety characteristics, and water resistance, emulsion explosives find widespread applications in tunnel excavation, mining, and building demolition.^4^ As water-containing explosives, they usually contain 8–15% water by weight. While this water content confers strong water resistance, it simultaneously acts as an inert component that reduces the overall detonation energy of the explosive.^5^ In scenarios such as bench deep-hole blasting, medium-hard rock blasting, and underwater blasting, the insufficient energy per unit volume of emulsion explosives becomes a particularly critical limitation. Hence, enhancing the detonation energy per unit volume without compromising the fundamental properties of emulsion explosives is of significant importance for the development of high-energy emulsion explosives.

The introduction of micro-bubbles into emulsion explosives through sensitization can, to some extent, enhance their detonation energy.^6^ This enhancement is attributable to the formation of “hot spots” within the explosive matrix. Upon excitation by a shock wave, the explosive material surrounding these hot spots is activated, initiating the detonation reaction.^7^ Conventional chemical sensitization involves adding sodium nitrite (NaNO_2_) to the explosive matrix, which subsequently generates nitrogen gas (N_2_) bubbles to sensitize the emulsion explosive. However, due to the relatively slow reaction rate of NaNO_2_ sensitization, the resulting sensitizing bubbles exhibit non-uniform particle sizes. This leads to a higher porosity within the explosive. Furthermore, because the oxidizer and fuel reside in distinct phases, emulsion explosives demonstrate significant non-ideal detonation behavior.^8^ This not only prevents the complete release of the explosive's energy but also exacerbates the generation of greenhouse gases, such as nitrogen oxides (NO_*x*_) and carbon monoxide (CO).

Another primary approach to enhance the detonation energy of emulsion explosives involves optimizing their energy release efficiency through the incorporation of high-energy additives. Research indicates that introducing energetic material components can significantly improve the system's detonation performance. The addition of RDX^9^ and composition B^10^ has been shown to linearly increase the detonation velocity and energy parameters of emulsion explosives, with the energy gain effect exhibiting a positive correlation with the additive content. Similarly, the introduction of metal powders (*e.g.*, Al,^11^ Ti^12^) can markedly enhance the specific shock wave energy, specific bubble energy, and total energy in underwater explosions through secondary combustion reactions. Notably, hydrogen-based energy carriers have attracted Widespread attention due to their high combustion enthalpy and environmentally friendly characteristics. Cheng *et al.* systematically revealed the dual mechanism of metal hydrides (*e.g.*, MgH_2_,^13,14^ TiH_2_ (ref. 15 and 16)): they act as sensitizers to lower the initiation threshold while simultaneously enhancing the detonation energy density by releasing active hydrogen upon decomposition. However, the associated decrease in initial decomposition temperature (Δ*T* = 19.9 °C) and apparent activation energy (Δ*E* = 10.4 kJ mol^−1^) imposes a significant negative impact on the thermal stability of the system.^17^ To overcome this drawback, Chen *et al.*^18^ and Wang *et al.*^19^ innovatively employed high-pressure containment technology to store active H_2_ within glass microballoons. Experimental results confirmed that this structure can simultaneously achieve energy enhancement and safety control—the hydrogen released upon the rupture of the microballoons supports sustained detonation wave propagation through chain combustion reactions, leading to an exponential growth trend in detonation velocity and energy output with increasing H_2_ loading. Although the aforementioned studies confirm that high-energy additives can produce a sensitization-energy enhancement synergy, a high additive content significantly alters the physicochemical properties of the emulsion explosive. Furthermore, the mechanism of energetic additives relies on introducing external energy; they fail to ameliorate the non-ideal detonation characteristics inherent to emulsion explosives effectively. This results in a lower utilization efficiency of the explosive's intrinsic energy, leading to resource wastage.

Based on the characteristic of oxygen as an efficient oxidizer, its energy-releasing reaction with reductive substances follows the theory of molecular thermal motion: elevated temperatures accelerate the movement of reactant molecules, significantly enhancing the chemical reaction rate and energy release efficiency. Within an explosion reaction system, the high-temperature and high-pressure environment generated by an air blast can induce secondary reactions between explosion products and atmospheric oxygen,^20^ achieving a multiplicative effect on energy release. Existing research has introduced bound oxygen into emulsion explosive systems through glass microballoon oxygen storage technology, confirming the enhancing effect of oxygen content on explosion power.^19^ However, the technical approach of directly sensitizing emulsion explosives with free-state oxygen has not been reported. Therefore, this study innovatively proposes an *in situ* oxygen-generation sensitization technology based on an M foaming agent. This technology not only addresses the issue of non-uniform sensitizing bubble size in traditional emulsion explosives but also, through the energy enhancement effect of oxygen, promotes complete detonation of the explosive. This reduces the non-ideal detonation characteristics of emulsion explosives and increases their detonation energy.

This study systematically investigates the feasibility of sensitizing emulsion explosives with oxygen generated from the reaction of an M foaming agent, along with the resulting detonation and thermal decomposition characteristics. Microscopic observation techniques and density measurements were employed to analyze the bubble formation behavior within the explosive samples. The energy release during detonation was studied by combining detonation velocity measurements with air blast shock wave testing. Furthermore, the thermal decomposition characteristics were examined through TG-DSC experiments by fitting the kinetic parameters of the explosive's thermal decomposition.

## 2 Experimental materials and methods

### 2.1 Materials and instruments

The emulsion matrix was prepared in our laboratory. Its composition and content are listed in Table 1. The raw materials included ammonium nitrate (NH_4_NO_3_, supplied by Henan Jinkai Chemical Investment Holding Group Co., Ltd), sodium nitrate (NaNO_3_, supplied by Yunnan Jingrui Technology Co., Ltd), compound oil phase (CO, Prepared by the laboratory), sodium nitrite (NaNO_2_, supplied by Shanghai Titan Scientific Co., Ltd), citric acid (monohydrate, supplied by Chengdu Kelong Chemical Co., Ltd), and the M foaming agent, which was prepared in our laboratory. The M foaming agent is a proprietary composite formulation whose primary active component is hydrogen peroxide (H_2_O_2_), designed to decompose and release oxygen gas upon thermal activation.

**Table 1:** Composition and content of the emulsion matrix

Constituent	NH_4_NO_3_	NaNO_3_	H_2_O	CO		
				C_18_H_38_	C_12_H_26_	C_24_H_44_O_6_
Content (%)	75	10	8.5	3.5	1	2

### 2.2 Experimental procedure

#### 2.2.1 Sample preparation

According to the formulation provided in Table 1, NH_4_NO_3_ and NaNO_3_ were mixed with water and then emulsified with the compound oil phase (CO) at an elevated temperature using a high-shear emulsifier to obtain the base emulsion matrix.

Sodium nitrite (NaNO_2_) and citric acid monohydrate were separately dissolved in deionized water to prepare aqueous solutions with mass concentrations of 50% and 25%, respectively. After the base emulsion matrix cooled to approximately 50 °C, the sodium nitrite solution and the citric acid solution were sequentially added to it according to a mass ratio of matrix : sensitizer : sensitization aid = 99.6 : 0.2 : 0.2. The mixture was mechanically stirred for 2 minutes until homogeneous. The resulting conventional emulsion explosive sample was labeled as Sample 0. Following the proportions listed in Table 2, Samples 1, 2, 3, and 4 were prepared by incorporating the M foaming agent into the base emulsion matrix using the method described above.

**Table 2:** Composition and mass ratio of the emulsion explosive samples

Constituent content	Latex matrix	NaNO_2_ (50%)	C_6_H_8_O_7_ (25%)	M foaming agent
Sample number				
0	99.6	0.2	0.2	
1	99.6			0.4
2	99.2			0.8
3	98.8			1.2
4	98.4			1.6

Furthermore, we emphasized that all explosive formulation, handling, and testing procedures were conducted in strict compliance with the safety regulations of Jianghan University and Anhui University of Science and Technology, and under the appropriate legal frameworks governing energetic materials research in China.

#### 2.2.2 Detonation velocity test

**Fig. 1:**
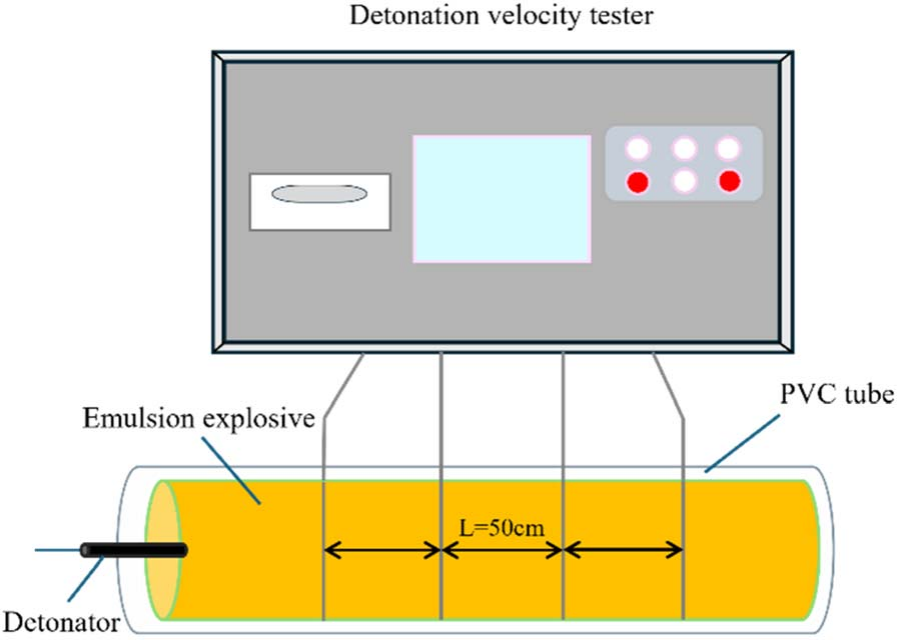
Detonation velocity testing experiment.

The detonation velocity of the emulsion explosives was measured using the electric probe method in conjunction with an intelligent five-segment detonation velocimeter. The experimental setup is illustrated in Fig. 1. For the test An apparatus, a PVC tube with specifications of *Φ* 40 mm (outer diameter) × 4 mm (wall thickness) × 300 mm (length), was used as the charge container. Approximately 500 g of the emulsion explosive sample was densely packed into the tube. An electronic detonator was installed at the initiation end. To ensure stable detonation wave propagation, the first target wire was positioned 70 mm from the initiation end. When the detonation wave front reaches a target wire, the high-temperature, high-pressure reaction zone instantly melts and breaks the wire conductor, causing a sharp decrease in its electrical resistance. By measuring the time difference between the electrical signal changes generated by adjacent target wires and combining this with the preset distance between them, the average propagation velocity of the detonation wave within the test section can be calculated.

#### 2.2.3 Air blast test

**Fig. 2:**
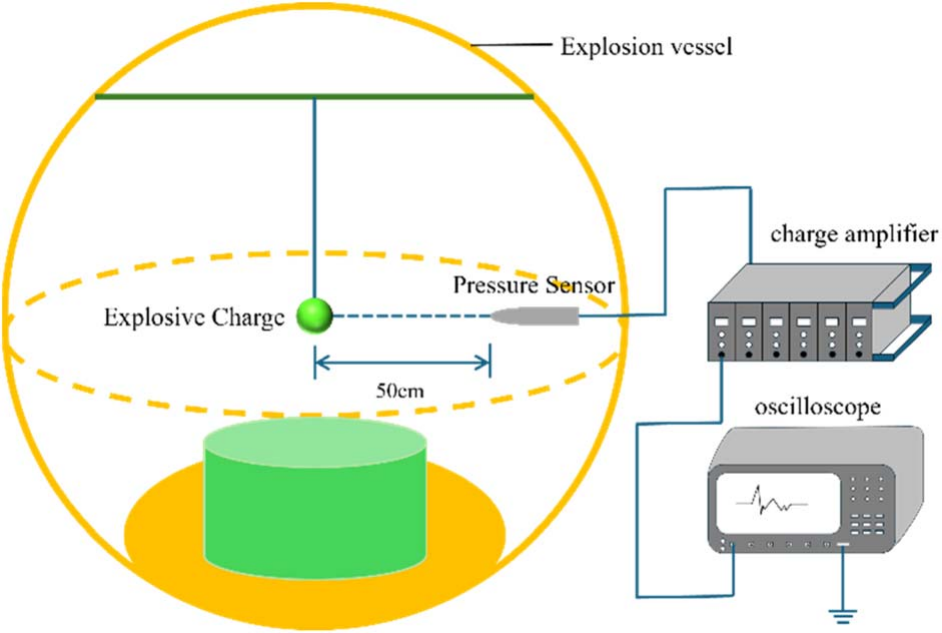
Air explosion testing experiment.

The detonation energy of the emulsion explosive samples was quantitatively characterized through air blast tests, primarily by measuring three key parameters: the peak shock wave overpressure, the positive phase duration, and the positive impulse. The experimental setup is shown in Fig. 2. A 50 g charge of the emulsion explosive was encapsulated in a polyvinyl chloride (PVC) film to form a spherical charge. This charge was positioned at the geometric center of the explosion chamber using a suspension mechanism, ensuring the center of the charge was 100 cm above the ground and maintained a horizontal distance of 50 cm from the CY-YD-202 piezoelectric pressure sensor. The sensor converted the shock wave pressure signal into an electrical signal *via* a charge amplifier. This signal was then recorded as a voltage–time curve by an oscilloscope. Processing this curve yielded the pressure-time history curve of the air blast.

#### 2.2.4 TG-DSC test

Thermogravimetric and Differential Scanning Calorimetry (TG-DSC) tests were conducted using a simultaneous thermal analyzer (TGA/DSC 3+, Mettler Toledo, Switzerland) to investigate the thermal properties of the base emulsion matrix, the sodium nitrite-sensitized emulsion explosive sample (Sample 0), and the M foaming agent-sensitized explosive samples (Samples 1–4). For the test, approximately 6 mg of the sample was weighed. The experiment was performed under a nitrogen atmosphere with a gas flow rate of 20 mL min^−1^. The heating rates were set to 5, 10, 15, and 20 K min^−1^, respectively, over a temperature range from 25 to 350 °C, and the corresponding TG-DSC curves were recorded.

## 3 Results and discussion

### 3.1 Sample characterization

After adding sodium nitrite (NaNO_2_) to the emulsion matrix, it reacts with ammonium nitrate (NH_4_NO_3_) present in the matrix to form ammonium nitrite (NH_4_NO_2_). The unstable NH_4_NO_2_ subsequently decomposes into nitrogen gas (N_2_) and water (H_2_O), thereby achieving sensitization by introducing gas bubbles into the matrix. The reaction mechanism is shown in the following equation:

1 NaNO_2_ + NH_4_NO_3_ → NH_4_NO_2_ + NaNO_3_

2 NH_4_NO_2_ → N_2_ + H_2_O

In contrast, when the M foaming agent is incorporated into the matrix, it undergoes rapid decomposition upon thermal activation, generating oxygen gas (O_2_) to achieve sensitization. The reaction mechanism is shown in eqn (3):

3 H_2_O_2_ → O_2_ + H_2_O

Based on the stoichiometric ratio and relative molecular masses, the theoretical gas production volume upon complete reaction should be approximately equal when the mass ratio of the M foaming agent to sodium nitrite (NaNO_2_) is 2 : 1. Therefore, in this experiment, Sample 1 (containing 0.4% M foaming agent) and Sample 0 were selected as the control groups for comparison.

The density of the laboratory-prepared base emulsion matrix sample was 1.39 g cm^−3^. The densities of the emulsion explosive samples sensitized with different agents are shown in Table 3. The data indicate that the addition of either NaNO_2_ or the M foaming agent significantly reduced the density of the base matrix. This confirms that chemical reactions occurred, generating gas bubbles, and verifies the feasibility of using the M foaming agent as a sensitizer for emulsion explosives. Furthermore, as the content of the M foaming agent increased, the sample density decreased, indicating a corresponding increase in the volume of oxygen gas (O_2_) produced by its reaction. This means the content of sensitizing gas per unit volume of the explosive increased. Comparing Sample 0 and Sample 1, the density of Sample 1 was higher than that of Sample 0. This is primarily attributed to the lower relative molecular mass of nitrogen gas (N_2_, 28 g mol^−1^) compared to oxygen gas (O_2_, 32 g mol^−1^), which results in a larger bubble volume for an equivalent mass of N_2_ gas. Additionally, gas bubbles represent a thermodynamically unstable system. To minimize surface free energy, bubbles tend to coalesce, reducing their specific surface area. Nitrogen bubbles are more prone to this coalescence, leading to the formation of larger bubbles and a greater overall sample volume, consequently resulting in a lower density.

**Table 3:** Density and OB of the emulsion explosive samples

Sample number	0	1	2	3	4
Density (g cm^−3^)	1.22	1.28	1.25	1.21	1.18
OB (g g^−1^)	−0.638	−0.637	−0.634	−0.631	−0.629

Beyond the physical sensitization effect governed by bubble morphology, the chemical nature of the sensitizing gas, specifically, the introduction of active oxygen, is anticipated to directly influence the explosive's energy release efficiency. This influence is fundamentally captured by the oxygen balance (OB) of the formulation. Therefore, the OB for each sample was calculated to quantitatively assess this chemical effect. The OB is calculated by the following formula:4
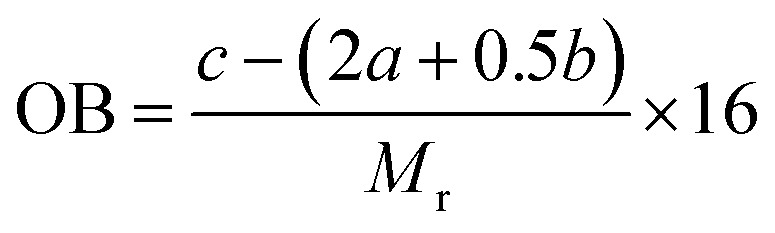
where *M*_r_ represents the relative molecular mass of a certain substance, and *a*/*b*/*c* respectively denote the number of carbon, hydrogen, and oxygen atoms contained in this substance. The calculation results of OB are shown in Table 3.

**Fig. 3:**
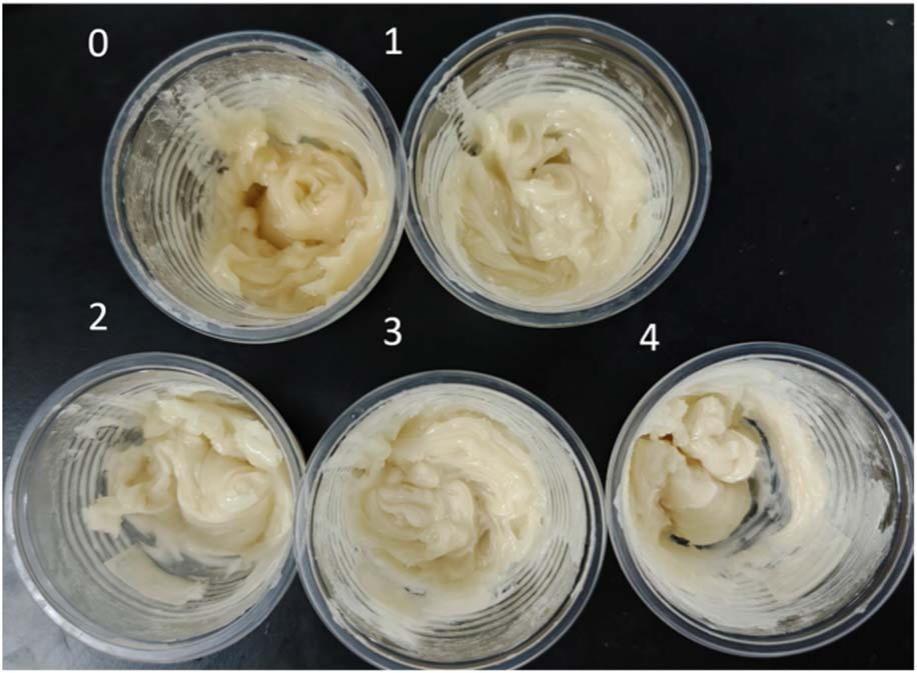
Physical photographs of the explosive samples.

Furthermore, Fig. 3 shows a photograph of the emulsion explosive samples. The base emulsion matrix typically exhibits a milky white appearance. However, Sample 0 in Fig. 3 displays a distinct yellowish-green color. This coloration is attributed to an undesired side reaction between sodium nitrite (NaNO_2_) and ammonium nitrate (NH_4_NO_3_) under high temperature and acidic conditions, which produces reddish-brown nitrogen dioxide (NO_2_) gas. The dissolution of this NO_2_ gas into the emulsion matrix results in the yellowish tint observed in the explosive sample. The reaction mechanism is shown in the following equation:

5 H^+^ + NaNO_2_ → Na^+^ + HNO_2_

6 HNO_2_ → NO + NO_2_ + H_2_O

**Fig. 4:**
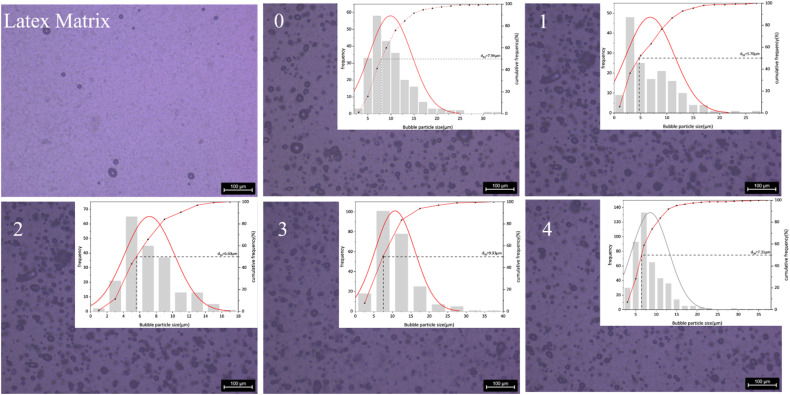
The microscopic morphology of the explosive sample and the size distribution of the sensitizing bubbles.

The oxygen gas (O_2_) produced by the reaction of the M foaming agent is inherently colorless. Its dissolution into the emulsion matrix allows the samples to retain a white appearance. Consequently, from Sample 1 to Sample 4, the color of the explosive samples progressively becomes whiter, providing further evidence that the M foaming agent can effectively function as a sensitizer for emulsion explosives. Fig. 4 shows the micromorphological characteristics of the base emulsion matrix and the emulsion explosive samples under different sensitization conditions.

As shown in Table 4 and Fig. 4, a significant difference in bubble morphology is observed when comparing Sample 0 (sensitized with NaNO_2_) and Sample 1 (sensitized with the M foaming agent). Quantitative analysis indicates that although the M foaming agent produces microbubbles with a smaller mean diameter than those in the NaNO_2_-sensitized sample, its bubble population exhibits poorer size uniformity, as reflected by a higher polydispersity index. This morphological disparity originates from fundamental differences in sensitization reaction kinetics. The rapid decomposition of the M foaming agent produces oxygen at a higher rate than the gas generation reaction in NaNO_2_ sensitization, resulting in rapid, massive, and multi-point nucleation of O_2_ bubbles that are effectively trapped by the highly viscous oil-phase matrix. Further analysis of Samples 1 through 4 reveals a non-monotonic relationship between M foaming agent content and bubble morphology. The mean bubble diameter does not vary linearly with concentration; instead, it initially increases from 6.83 μm (Sample 1) to 10.71 μm (Sample 3) before decreasing to 8.55 μm (Sample 4). Concurrently, the bubble population uniformity, as represented by the polydispersity index (PDI), also follows a complex pattern, with Sample 2 exhibiting the most uniform distribution (PDI = 0.18). This morphological evolution suggests a dynamic competition between bubble nucleation and coalescence processes. At moderate concentrations, increased gas production may initially promote bubble coalescence, leading to larger average sizes. However, as the sensitizer content continues to rise, the dramatically increased nucleation site density eventually dominates, resulting in a larger number of finer bubbles. This complex interplay ultimately leads to the observed non-monotonic trend in both bubble size and distribution uniformity across the concentration series.

**Table 4:** Particle size distribution of sensitization bubbles in explosive samples

Sample number	Mean particle size (μm)	Median particle size (μm)	Polydispersity index (PDI)
0	9.91	8.83	0.21
1	6.83	5.71	0.45
2	7.17	6.60	0.18
3	10.71	9.93	0.25
4	8.55	7.31	0.28

### 3.2 Detonation velocity

Following detonator initiation, the surface layer of the emulsion explosive is activated. Gas bubbles function as “hot spots” that undergo adiabatic compression under shock wave loading, triggering detonation reactions in adjacent explosive material. This energy release sustains the leading shock front, ultimately establishing stable detonation wave propagation through the explosive medium. Detonation velocity (*V*_D_) denotes the propagation speed of this detonation wave. According to Chapman–Jouguet (C–J) theory:^21^7
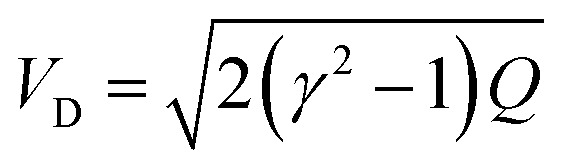
where *V*_D_ is the detonation velocity (m s^−1^) of emulsion explosive, *γ* is the adiabatic index of explosion products, and *Q* is the energy released by the explosion reaction of the explosive (J kg^−1^).

**Fig. 5:**
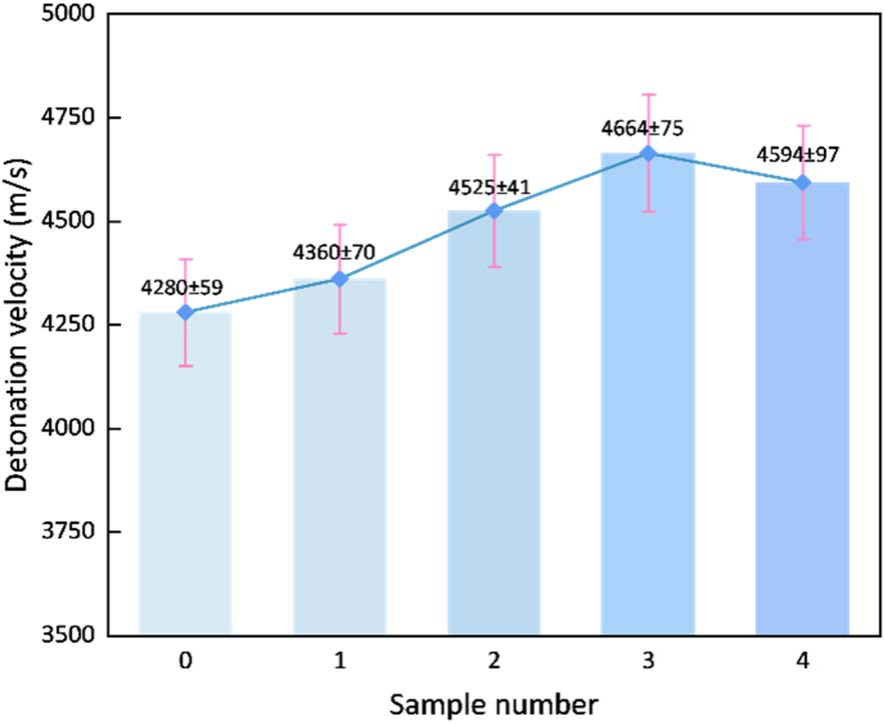
Detonation velocity of the explosive samples.

Beyond their role as “hot spots”, sensitizing bubbles enhance detonation performance through an additional physical mechanism. Under shock wave compression, the rapid collapse and fragmentation of these bubbles generate gas micro-jets, which significantly intensify turbulent mass transfer between the explosive matrix and the gas phase.^22^ This process accelerates the diffusion-controlled combustion reaction, thereby facilitating the stabilization and propagation of the detonation wave. As illustrated in Fig. 5, the detonation velocity is strongly influenced by both the type and content of sensitizing bubbles. In the conventional NaNO_2_-sensitized system, the adiabatic compression of bubbles releases inert N_2_ gas. Although the resulting micro-jets improve local mixing, the nitrogen itself does not participate in exothermic reactions. Thus, the energy supporting the leading shock wave is limited to that released from the base explosive components, resulting in only moderate detonation velocity enhancement. In contrast, the micro-bubbles produced by the M foaming agent release highly reactive O_2_ upon collapse. It is plausible that during the turbulent mixing process, this oxygen may engage in secondary oxidation reactions with combustible species, potentially including incompletely oxidized intermediates such as carbon monoxide (CO).^23,24^ If such reactions occur, the additional chemical energy released *via* this pathway could continuously reinforce the detonation wave, potentially contributing to the observed increase in detonation velocity. Moreover, the fine bubble size and homogeneous spatial distribution in the M foaming agent-sensitized system (Fig. 4) increase the density of effective reaction sites and optimize the wave propagation trajectory.

Furthermore, Fig. 5 reveals a non-monotonic effect of the M foaming agent content on the detonation velocity. This trend can be interpreted through the coupled influence of chemical composition and physical structure on the detonation process. As the additive amount increased from 0.2% to 0.6%, two synergistic effects contributed to the rising detonation velocity: firstly, the introduction of active oxygen mitigated the intrinsic negative oxygen balance of the emulsion matrix (as shown in Table 3), promoting more complete oxidation of the fuel components and thereby enhancing the chemical energy release;^25^ secondly, the formation of a fine and uniform bubble population (as quantified in Section 3.1) increased the density of effective “hot spots”, improving the initiation efficiency of the detonation reaction.

However, when the content exceeded 0.6%, the physical dilution effect became dominant. Observation shows that a larger mean bubble diameter and a higher bubble content result in a decrease in the bulk density of the explosive. This lower density, coupled with a coarsened bubble structure, is known to broaden the detonation reaction zone.^26^ A wider reaction zone increases the susceptibility of the detonation wave to energy losses through lateral rarefaction, particularly in a charge of finite diameter. Although the oxygen balance continued to improve, this positive chemical effect was outweighed by the enhanced wave-front dissipation, ultimately leading to the observed decline in detonation velocity. Thus, the performance peak at 0.6% represents an optimal compromise between the beneficial chemical (oxygen balance) and physical (“hot spot” density) factors and the detrimental physical effect of reaction zone broadening.

### 3.3 Air blast

**Fig. 6:**
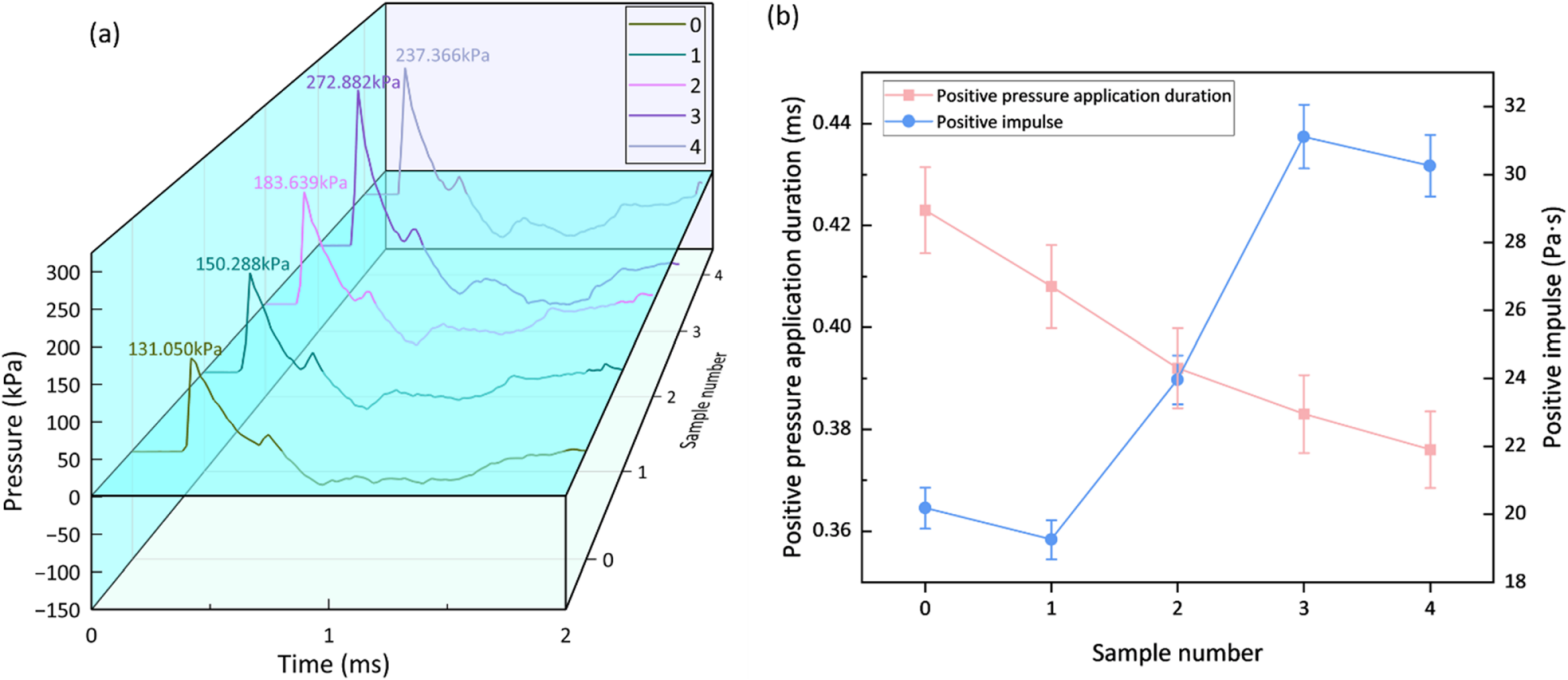
Air explosion parameters of explosive samples, (a) peak overpressure, (b) positive pressure application duration and positive impulse.

When an explosive detonates in a free field, its detonation products rapidly compress the surrounding air, forming an air blast shock wave. The shock wave parameters, such as the peak overpressure, positive phase duration, and impulse, can serve as the basis for assessing damage to objects and thus act as a Measure of the energy released by the explosion. Table 5 presents the shock wave parameters obtained from the air blast tests. Fig. 6(a) shows the peak overpressure (*P*_m_) for each sample, and Fig. 6(b) illustrates the trends of the positive phase duration (*t*_+_) and positive impulse (*I*_+_) with the type and content of the sensitizing agent. The positive impulse is calculated as the integral of the overpressure over the positive phase duration:8
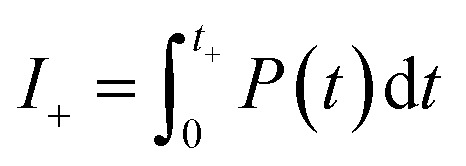
where *P*(*t*) is the positive pressure at time *t*.

**Table 5:** Shock wave parameters of the sample's air explosion experiment

Sample number	Peak overpressure (*P*_m_)/kPa	Positive pressure application duration (*t*_+_)/ms	Positive impulse (*I*_+_)/pa s
0	131.050	0.423	20.174
1	150.288	0.408	19.242
2	183.639	0.392	23.958
3	272.882	0.383	31.115
4	237.366	0.376	30.261

During an air blast event, the peak overpressure (*P*_m_) characterizes the transient energy release intensity of the emulsion explosive, while the positive phase duration (*t*_+_) and positive impulse (*I*_+_) reflect the sustainability of the energy release. The data in Table 5 indicate that the *P*_m_ of the M foaming agent-sensitized system (150.288 kPa) is significantly higher than that of the conventional NaNO_2_-sensitized system (131.050 kPa), representing an increase of 14.68%. This phenomenon originates from the dual energy contribution mechanism of the active oxygen released by the M foaming agent's reaction. One portion of the O_2_ directly participates in “hot spot” formation, initiating the initial detonation. Another portion, released upon the collapse and fragmentation of bubbles, undergoes intense combustion reactions with the combustible components (*e.g.*, fuel oil) in the emulsion matrix through turbulent mixing. This process continuously compensates for the energy dissipation of the leading shock wave, thereby enhancing *P*_m._

Concurrently, the small and uniformly distributed micro-bubbles in the M foaming agent-sensitized system significantly increase the detonation reaction rate and the concentration of energy release, further synergistically enhancing both the detonation velocity and the peak overpressure. This is corroborated by the pressure-time history curves in Fig. 6(a), where the curve for the sodium nitrite-sensitized Sample 0 is smoother, while those for the M foaming agent-sensitized Samples 1–4 are relatively steeper. It is noteworthy that the conventional NaNO_2_-sensitized system, due to its negative oxygen balance and non-ideal detonation characteristics, tends to generate significant amounts of incomplete combustion products such as carbon monoxide (CO) and nitrogen oxides (NO_*x*_).^27,28^ In contrast, the active oxygen released within the product expansion zone of the M foaming agent system partially mitigates the formulation's negative oxygen balance, thereby promoting more complete oxidation reactions that reduce the generation of pollutant gases such as carbon monoxide (CO) and nitrogen oxides (NO_*x*_), while simultaneously releasing additional chemical energy through these secondary oxidation processes.^29,30^ This modification promotes more complete detonation reactions, thereby enhancing the overall explosive energy output and leading to a more efficient and thorough detonation process. This mechanism was validated in gradient experiments where the M foaming agent content increased from 0.2% to 0.6%: *P*_m_ surged from 150.288 kPa to 272.882 kPa (an increase of 81.57%), revealing the potential for improving the non-ideal detonation characteristics of emulsion explosives.

However, when the amount of the M foaming agent exceeded a threshold, the system's capacity to utilize the additional oxygen becomes saturated. The fuel-rich matrix cannot fully oxidize the surplus oxygen, which then acts as an inert diluent. This aligns with the fundamental principle that for a given fuel/oxidizer system, there exists an optimal oxygen balance for maximum energy release.^31^ Further oxygen addition beyond this point leads to a decrease in the energy density per unit volume. Besides, an excessive volume of gas bubbles can lead to a widening of the detonation reaction zone. A wider reaction zone makes the detonation wave more vulnerable to energy losses through lateral rarefaction, especially in charges of finite diameter. This effect can destabilize the detonation wave and reduce its velocity and pressure, as described by the Eyring equation and related models for non-ideal explosives.^26,32^ Coupled with the system's slightly negative oxygen balance, which prevented the effective utilization of the surplus oxygen, and the rarefaction wave effect that attenuated shock wave propagation efficiency, these factors ultimately led to a decrease in *P*_m_.

As shown in Fig. 6(b), the positive phase duration (*t*_+_) and positive impulse (*I*_+_) of the explosive samples exhibit significantly divergent evolutionary trends. The value of *t*_+_ decreased monotonically with increasing M foaming agent content, whereas *I*_+_ displayed a non-monotonic variation, reaching a maximum at an M foaming agent content of 0.6%. This phenomenon stems from fundamental differences in the dynamics of sensitizing bubbles and the modes of energy release. Compared to the NaNO_2_-sensitized system, the M foaming agent reaction produces micro-bubbles of smaller size, leading to an increased O_2_ release rate and an accelerated secondary combustion reaction process. Furthermore, the increased curvature of the bubble interfaces enhances turbulent mixing, causing energy to be released more concentratedly within a shorter timeframe. Although this high-speed energy release characteristic significantly enhances the peak overpressure (as seen in Fig. 6(a)), it also intensifies the dissipative effect of rarefaction waves on the expanding products, resulting in a shortened *t*_+_. Notably, increasing the M foaming agent content further amplifies this effect, manifesting as a graded decrease in *t*_+_. Data analysis further indicates that when the M foaming agent content was ≤0.6%, the rise in *P*_m_ significantly offset the reduction in *t*_+_, driving the growth of *I*_+_. Conversely, when the content exceeded 0.6%, the sharp decline in *P*_m_ (a 15.0% decrease at 0.8% content compared to 0.6%) synergized with the continued shortening of *t*_+_, creating a combined attenuation effect that ultimately caused *I*_+_ to decrease. This suggests that *I*_+_ is primarily dominated by *P*_m_.

### 3.4 Thermal decomposition characteristics

**Fig. 7:**
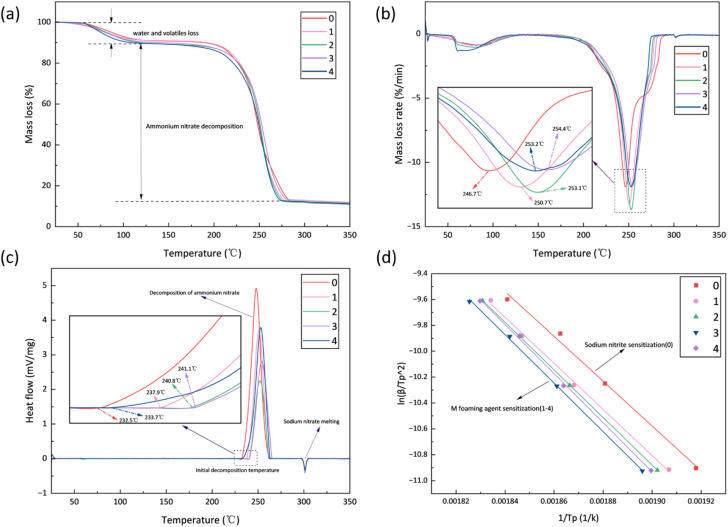
(a) TG; (b) DTG curves of the explosive samples at a heating rate of 5 K min; (c) DSC curves of the explosive samples at a heating rate of 5 K min; (d) Kinetic parameter curves obtained by Kissinger fitting based on the peak temperatures of the DSC curves.

The thermal decomposition behavior was investigated using TG-DSC analysis at a heating rate of 5 K min^−1^ to capture detailed decomposition characteristics. Fig. 7 presents the TG and corresponding DTG curves for all samples. The TG curves show similar two-stage decomposition patterns for all formulations, indicating that the M-foaming agent does not fundamentally alter the decomposition pathway.

All samples exhibited comparable mass loss profiles: an initial 10% mass loss between 50–120 °C corresponding to moisture evaporation, followed by the main decomposition stage (80% mass loss) between 180–285 °C, attributed to ammonium nitrate decomposition and redox reactions. The residual mass showed minimal variation among samples. The DTG curves revealed significant differences in thermal stability. The NaNO_2_-sensitized sample (Sample 0) showed a peak decomposition temperature at 246.7 °C, while M-foaming agent sensitized samples (Samples 1–4) exhibited elevated peak temperatures of 250.7 °C, 253.1 °C, 254.4 °C, and 253.2 °C, representing increases of 4.0–7.7 °C. This consistent shift to higher temperatures demonstrates the thermal stabilization effect of the M-foaming agent. DSC analysis (Fig. 7c) further confirmed these findings, showing exothermic peaks in the 230–270 °C range corresponding to ammonium nitrate decomposition. The M-foaming agent sensitized samples displayed higher initial decomposition temperatures (233.7–241.1 °C) compared to the NaNO_2_-sensitized sample (232.5 °C), with increases of 1.2–8.6 °C. The consistent elevation of both peak and initial decomposition temperatures across multiple analysis methods confirms the enhanced thermal stability imparted by the M-foaming agent.

To gain deeper insight into the influence of different sensitizers on the thermal decomposition behavior of emulsion explosives, this study performed kinetic analysis of the thermal decomposition data using both the Kissinger method and the Ozawa method. These two methods utilize characteristic temperature data obtained at different heating rates (5, 10, 15, and 20 K min^−1^): the Kissinger method employs the peak temperature (*T*_p_) of the decomposition process, whereas the Ozawa method is based on the temperature corresponding to a fixed conversion rate (50% mass loss). The kinetic equations used for the Kissinger method and the Ozawa method are shown in eqn (9) and (10), respectively. Apparent activation energies and other kinetic parameters for the thermal decomposition of each sample were calculated through linear fitting of the experimental data, with the detailed results presented in Table 6.9
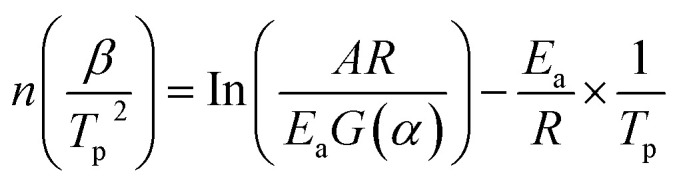
10
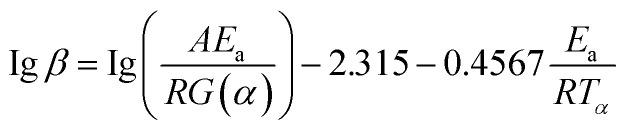
where *β* represents the heating rate (K min^−1^); *A* represents the pre-exponential factor (s^−1^); *T* represents the reaction temperature (K); *E*_a_ represents the apparent activation energy (kJ mol^−1^); *R* represents the ideal gas constant (*R* = 8.314 J mol^−1^ K^−1^); *G*(*α*) represents the integral mechanism function, where *α* denotes the conversion, *T*_*α*_ represents the temperature at which the conversion rate is *α*.

**Table 6:** Thermal decomposition kinetic parameters of the emulsion explosive obtained by the K method and O method

	*T*_p/_ *T*_50%_ (K)				Kissinger method		Ozawa method	
	5 (K min^−1^)	10 (K min^−1^)	15 (K min^−1^)	20 (K min^−1^)	*E*_a_ (kJ mol^−1^)	*R* ^2^	*E*_a_ (kJ mol^−1^)	*R* ^2^
0	521.4/519.2	531.7/530.8	536.9/535.2	543.2/541.5	144.3	0.993	141.0	0.991
1	524.4/520.2	535.3/532.3	541.4/537.8	545.2/541.2	148.3	0.997	144.1	0.990
2	525.7/521.2	535.8/533.8	541.7/537.9	546.2/543.5	152.9	0.999	141.3	0.988
3	527.4/523.4	537.3/536.5	542.9/540.2	547.8/544.8	155.6	0.999	145.5	0.980
4	526.4/522.1	536.5/535.0	541.7/538.8	546.5/543.5	157.9	0.997	145.2	0.983

As shown in Fig. 7(d) and Table 6, distinct differences are observed in the apparent activation energy (*E*_a_) between the emulsion explosive samples sensitized with NaNO_2_ and those sensitized with the M foaming agent. The kinetic parameters obtained from both the Kissinger and Ozawa methods (at 50% conversion) consistently demonstrate higher thermal stability for the M foaming agent-sensitized systems. Specifically, the Kissinger method reveals a clear increasing trend in *E*_a_ values from 144.3 kJ mol^−1^ (Sample 0) to 157.9 kJ mol^−1^ (Sample 4) with increasing M foaming agent content, while the Ozawa method at 50% conversion shows *E*_a_ values fluctuating between 141.0-145.5 kJ mol^−1^, still maintaining higher values than the reference sample.

The kinetic analysis provides crucial insights into the different decomposition pathways induced by the two sensitization mechanisms. For the NaNO_2_-sensitized system (Sample 0), the relatively low *E*_a_ values are consistent with the known catalytic effect of nitrogen oxides (NO_*x*_),^33–35^ which act as radical initiators that significantly promote ammonium nitrate decomposition, thereby lowering the overall apparent activation energy. In contrast, the M foaming agent system exhibits significantly different kinetic behavior. The progressively increasing *E*_a_ values with additive content, as clearly demonstrated by the Kissinger method, suggest that the oxygen released from the M foaming agent alters the fundamental decomposition pathway. Rather than simply providing physical barrier effects, the oxygen actively participates in the decomposition chemistry, potentially through pre-oxidation of fuel components that generate more stable intermediates. This modified reaction pathway, requiring higher activation energy as indicated by both kinetic methods, effectively delays the decomposition process and raises the temperature required for the main exothermic reaction. The agreement between both kinetic methods in showing elevated *E*_a_ values, combined with the clear increasing trend revealed by Kissinger analysis, provides compelling evidence that the M foaming agent enhances thermal stability through chemical modification of the decomposition pathway. This mechanistic understanding explains the observed shifts to higher temperatures in both the DTG peak temperatures and DSC exotherms with increasing M foaming agent content.

Based on the TG-DSC results, kinetic parameter analysis, and the chemical composition of the emulsion matrix, a speculative decomposition pathway is proposed, highlighting the distinct role of the M foaming agent. The process initiates with physical transformations between 50–120 °C, involving the evaporation of water and volatilization of low-boiling oil-phase components. The primary exothermic stage occurs between 180–285 °C, where complex redox reactions take place. The kinetic analysis, particularly the increasing trend in apparent activation energy with temperature obtained through model-free methods, supports the hypothesis that in the M foaming agent-sensitized system, the released oxygen participates in early decomposition by promoting pre-oxidation of fuel components into more stable intermediates such as carboxylic acids or aldehydes. The primary reactions are schematically represented as:

11 Fuel (C_*x*_H_*y*_) + O_2_ → R-COOH/R-CHO

12 NH_4_NO_3_ + R-COOH/R-CHO → N_2_ + CO_2_ + H_2_O

These oxygenated intermediates (R-COOH/R-CHO) possess higher thermal stability than the original fuel molecules and act as radical scavengers. Their formation alters the decomposition pathway, increasing the overall energy barrier and effectively delaying the main exothermic reaction, thereby enhancing the thermal stability of the explosive matrix.

This reaction-path alteration—where oxygen competes with and suppresses radical-driven pathways typically catalyzed by NO_*x*_ in NaNO_2_-sensitized systems—introduces a higher energy barrier, rationalizing the observed increases in initial decomposition temperature and the shift of the main exotherm to higher temperatures. Above 290 °C, the endothermic peak corresponds to the melting of sodium nitrate (NaNO_3_),^17^ concluding the thermal transition sequence.

## 4 Conclusions

This study demonstrates that sensitizing emulsion explosives with the M foaming agent, utilizing the oxygen (O_2_) generated by its reaction to form sensitizing bubbles, not only enhances the detonation performance of the explosives but also improves their thermal stability to a certain extent. Using traditionally sodium nitrite (NaNO_2_)-sensitized emulsion explosives as a control group, the microstructure, detonation velocity, air blast performance, and thermal stability of the samples were systematically characterized. The following key conclusions were drawn, with the notable understanding that all experimental results characterize the initial properties of the freshly prepared samples and do not account for potential changes during long-term storage:

(1) The oxygen produced by the M foaming agent effectively sensitizes the emulsion explosives. Microscopic analysis revealed that compared to the NaNO_2_-sensitized sample, the M-foaming agent sensitized system exhibits a notable reduction in average bubble size and displays good uniformity. This optimizing effect becomes more pronounced with increasing content of the M-foaming agent.

(2) The detonation velocity of the explosive shows a non-monotonic trend with respect to the content of the M-foaming agent, first increasing and then decreasing. At an addition level of 0.6%, the detonation velocity reaches a maximum value of 4664 m s^−1^, which is 9.0% higher than that of the NaNO_2_-sensitized control group (4280 m s^−1^). This confirms the positive promoting effect of an appropriate amount of oxygen on detonation propagation.

(3) Air blast tests indicate that the M foaming agent sensitized system possesses a superior energy release rate and total energy output. The peak overpressure of the shock wave increases to 272.882 kPa (control group: 131.050 kPa), and the positive impulse is enhanced by 54%, highlighting the significant reinforcement of energy release dynamics due to the participation of oxygen in the reaction.

(4) Replacing NaNO_2_ with the M foaming agent shifts the thermal decomposition temperature of the emulsion explosive towards a higher range. The introduction of oxygen leads to an increase in the apparent activation energy and improves the thermal stability of the emulsion explosive.

## Conflicts of interest

There are no conflicts to declare.

## Supplementary Material

RA-015-D5RA06873H-s001

## Data Availability

The data supporting the findings of this study, including raw data from detonation velocity tests, air blast overpressure measurements, and TG-DSC analyses, are available within the main text and its supplementary information (SI) figures and tables. Any additional raw data files are available from the corresponding author (Quan Wang, wqaust@163.com) upon reasonable request. Supplementary information is available. See DOI: https://doi.org/10.1039/d5ra06873h.
